# Influence of Compression Loading on Acoustic Emission and Light Polarization Features in TeO_2_ Crystal

**DOI:** 10.3390/ma17143590

**Published:** 2024-07-20

**Authors:** Alexander Machikhin, Dmitry Chernov, Demid Khokhlov, Artem Marchenkov, Alexey Bykov, Yan Eliovich, Ivan Petrov, Timofey Balandin, Alexander Kren, Ilya Sergeev, Yuri Pisarevsky

**Affiliations:** 1 National Research University “Moscow Power Engineering Institute”, 14-1 Krasnokazarmennaya, 111250 Moscow, Russia; 2 Scientific and Technological Center of Unique Instrumentation of the Russian Academy of Sciences, 15 Butlerova, 117342 Moscow, Russia; 3 Mechanical Engineering Research Institute of the Russian Academy of Sciences, 4 M. Kharitonyevskiy Pereulok, 101990 Moscow, Russia; 4 National Research Centre “Kurchatov institute”, 1 Pl. Akademika Kurchatova, 123182 Moscow, Russia; 5 Institute of Applied Physics, National Academy of Sciences of Belarus, 16 Akademicheskaya, 220072 Minsk, Belarus

**Keywords:** acoustic emission, cross-polarization imaging, brittle materials, anisotropic crystals, compressive loading, tellurium dioxide

## Abstract

Monitoring the processes inside crystalline materials under their operating conditions is of great interest in optoelectronics and scientific instrumentation. Early defect detection ensures the proper functioning of multiple crystal-based devices. In this study, a combination of acoustic emission (AE) sensing and cross-polarization imaging is proposed for the fast characterization of the crystal’s structure. For the experiments, tellurium dioxide (TeO_2_) crystal was chosen due to its wide use in acousto-optics. Studies were performed under uniaxial compression loading with a simultaneous acquisition of AE signals and four polarized optical images. An analysis of the temporal dependencies of the AE data and two-dimensional maps of the light depolarization features was carried out in order to establish quantitative criteria for irreversible damage initiation and crack-like defect formation. The obtained results reveal the polarization image patterns and the AE pulse duration alteration specific to these processes, and they open up new possibilities for non-destructively monitoring in real-time the structure of optically transparent crystals under their operating conditions.

## 1. Introduction

One of the areas in which crystalline materials have become widespread is acousto-optics. The key material for the production of acousto-optical devices is currently tellurium dioxide (TeO_2_). Its unique properties allow for the building of small-sized and highly efficient devices for photonics and laser techniques [1,2,3,4,5,6,7]. These devices operate normally only under certain conditions, which are determined by the mechanical and optical characteristics of its components, among others. To predict the behavior of the crystal in real operating conditions, it is necessary to conduct many studies on its behavior under external stressors. Such studies, also referred to as flaw detection, make it possible to determine the operating conditions’ boundaries and to determine the overall failure tolerance of the device with a degree of certainty.

To solve the problem of crack detection in crystals, there are a few experimental methods that can be used based on X-ray radiation [8,9], polarized light [10,11] and other techniques.

In X-ray methods, the proportionality of the inter-atomic distance and radiation wavelength makes it possible to consider the inspected crystal as a three-dimensional diffraction lattice. The analysis of the X-ray diffraction data on such periodic structures allows us to understand the arrangement and type of atoms present, as well as to estimate the interatomic distance. Among all X-ray methods, X-ray diffraction techniques, including two- and three-crystal X-ray diffractometry, are most commonly used for crystal examination. The experimental results obtained with these techniques are compared with the calculations following from the dynamic theory of diffraction, allowing us to come to a clear conclusion about the structure of the crystal, including the presence of defects. These techniques are also used to study crystals under dynamic load—however, in general, the temporal resolution of such techniques is limited. This problem can be solved by using fast diffractometric techniques, such as adaptive X-ray optics [12,13]. In [14], studies on the influence of ultrasonic modulation on TeO_2_ crystals were conducted, which showed a complex picture of the evolution of the crystal structure under pressure, but, due to the impossibility of time-resolved measurements, no response was received on the nature of the changes observed. In [15], a new method of rapid X-ray diffractometry was proposed, which allowed the authors to observe structural changes in real-time, but the determination of the time of transition from the area of elastic deformation to irreversible structural change using only the proposed methodology remains difficult due to the limited brightness of the laboratory X-ray sources.

Optical microscopy provides observations of macroscopic defect formation in transparent brittle materials during mechanical loading [16,17,18]. Quantitative analysis of stress-induced artificial birefringence in isotropic materials allows us to conduct elastic stress assessments [19] with respect to the stress-optic law [20]. The application of photoelastic birefringent coatings facilitates shear stress monitoring for opaque materials [21,22]. The evolution of optical polarization features under external mechanical load in anisotropic optical crystals is guided by the complex combination of stress-induced birefringence and the intrinsic material anisotropy of the elastic and optical properties [11,23,24,25]. This hampers the application of optical techniques for studying anisotropic materials. One successful method for anisotropic crystal inspection is conoscopic interference pattern analysis [10,26,27]. With no external load applied, the conoscopic inspection indicates the inhomogeneity of the refractive indices formed during crystal growth and through mechanical post-growth processing. The precise inspection of thick crystalline specimens requires a stable and uniform laser illumination source [10]. At the same time, obtaining data on stress localization from fringe patterns during mechanical loading may be a challenging task.

Despite the obvious advantages of these methods, their use is significantly difficult when it is necessary to analyze the impact of the mechanical stress arising in the process of operation directly in ready-made devices. This issue may be solved by acoustic emission (AE) sensing, which is successfully used to study the condition of structural and functional materials, including the study of dynamic damage in alloys [28] and composite materials [29]. Different parameters can be used to estimate crystal defects using the AE method, both primary (e.g., amplitude (*u_m_*), duration (*t_imp_*), activity (*Ń*)) and complex, based on the calculations of certain functions (e.g., amplitude distribution (*u_m_*) and energy (*E_imp_*)). However, only complex AE data analysis allows us to determine informative AE parameters and their threshold values, indicating irreversible destruction in the inspected crystal. In [30,31,32], it was shown that, at the moment of formation and the development of irreversible damage in the crystal, a change in the parameters of the energy distribution and duration of AE pulses can be observed. This allows us to obtain analytical dependencies *E_imp_*(*t_imp_*) and *u_m_*(*E_imp_*), which correlate with the degree of damage to the crystals.

In this feasibility study, we propose to analyze AE data in TeO_2_ crystals under mechanical load, obtain quantitative criteria of defect appearance and confirm this by simultaneous cross-polarization imaging [33,34,35]. We introduce the criteria based on AE pulse duration for the evaluation of crystal damage degree instead of other AE signal parameters [36,37,38]. To simplify the polarization features’ extraction and conserve the most informative data on the angle and degree of light polarization, highlighting the structural inhomogeneities in crystal samples, we apply a linearly polarized illumination. The combination of these two techniques allows for real-time data acquisition and analysis, and thus the determination of the dependencies between the external load and the obtained AE and optical data.

## 2. Materials and Methods

### 2.1. Crystals

Sample preparation included crystal growth, orientation, quality control and processing. High-purity single α-TeO_2_ crystals were grown from a melt using the Czochralski method in a [110] direction (National Research Centre “Kurchatov institute”, Moscow, Russia). The crystal boule was 60 mm in diameter and had a 60 mm cylindrical part. The exact orientations of the crystallographic axes were determined using X-ray diffraction patterns. Quality control included spectral chemical analysis, dislocation density measurements by selective etching and inner defect detection by laser conoscopy [10]. Crystallinity and grain-free structure (high degree of perfection) were ensured by X-ray topography [39]. After the crystal was oriented and checked, samples were cut from it. Their facets were polished and coated to ensure a high level of flatness and optical transmission in the visible range.

**Figure 1:**
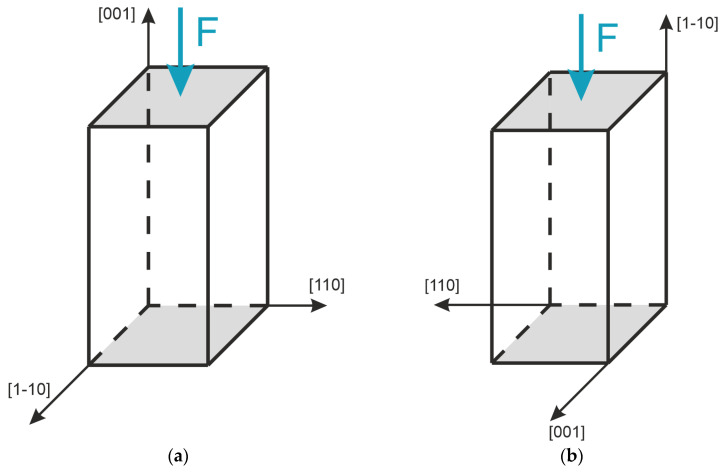
Configurations of the inspected samples of the first (**a**) and second (**b**) type.

In this study, we tested samples of two types (Figure 1). Both of them have dimensions of 10 ± 0.1 mm × 10 ± 0.1 mm × 20 ± 0.1 mm and four polished faces. The samples’ faces contacting the testing machine compression platens were beveled to prevent the undesired defect growth. Samples of the first type had polished and coated faces parallel to planes (110) and (1-10), providing optical imaging along the [110] and [1-10] axes and the application of compressive force *F* along the [001] axis. Specimens of the second type, with compressive force *F* applied along the [1-10] axis, were prepared for observation along the [110] and [001] axes.

The optical properties of α-TeO_2_ under normal conditions, including their polarization features, are well known [3,7]. Linearly polarized light conserves its polarization state when passing through a crystal in an arbitrary direction other than the vicinity of the optical axis [001], where gyrotropy is present.

Considering the mechanical properties of α-TeO_2_, it is necessary to pay attention to Young modulus and compressive strength. Young modulus significantly depends on direction and varies from 8.7 GPa to 112.4 GPa [40]. For the two mentioned specimen types, compressive Young modulus is 95.9 GPa along the [001] axis and 112.4 GPa along the [1-10] axis. The variability in compressive strength values reported in previous works are caused by different crystal growth techniques, initial specimen quality and the vulnerability of this brittle material with respect to the compression loading conditions. Furthermore, the compressive strength is expected not to exceed 150 MPa along the [001] axis and 120 MPa along the other one [41,42].

**Figure 2:**
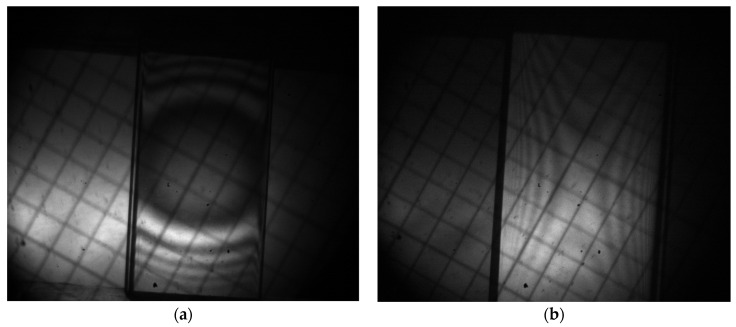
Examples of conoscopic interference patterns for two types of samples, i.e., prepared for compressive load along [001] (**a**) and [1-10] (**b**) axes.

Before the compressive tests, we inspected the crystalline samples with a standard conoscopic technique [10] in a static mode, i.e., without external mechanical load, to ensure the absence of structural (macroscopic) defects. The examples of conoscopic patterns for the specimens of both types are shown in Figure 2.

### 2.2. Experimental Setup

**Figure 3:**
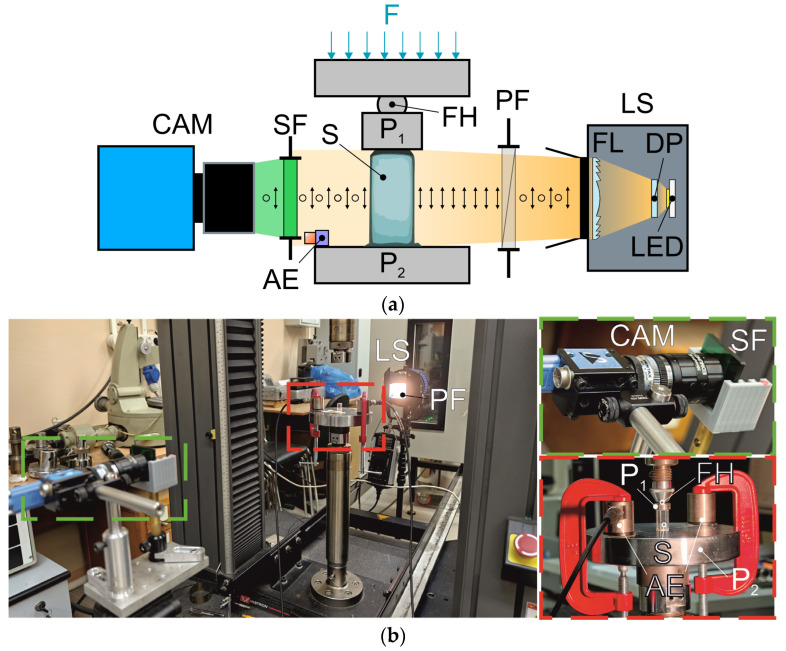
Scheme (**a**) and appearance (**b**) of the experimental setup: CAM—camera, P_1_ and P_2_—testing machine compression platens, AE—acoustic emission sensor, FH—floating hinge, SF—spectral filter, PF—polarizer, LS—light source, FP—Fresnel lens, DP—diffusing plate, LED—light-emitting diode, F—compression force applied to the press platen, and S—specimen.

To achieve simultaneous mechanical loading and a comprehensive visualization of the crystalline samples, we applied a complex experimental setup, which was a combination of an AE sensing system, a cross-polarization imager and a universal testing machine, (UTM) Instron 5982 (Instron GmbH, Darmstadt, Germany), providing the specimens with a uniaxial compressive load (Figure 3). For the precise control of external load and data acquisition synchronization, all three devices were connected to a single PC. The tested specimen was placed vertically on a 10 mm thick steel platen P_2_. Compressive force was applied by the moving upper platen P_1_, which was equipped with a floating hinge, providing the coincidence of compressive force F and the chosen crystallographic axis. Since paratellurite crystals have low plasticity, we used a floating hinge to avoid distortions during loading. During every single bench test, the upper UTM traverse moved downwards with a low loading rate (0.01 mm/min) to ensure a reliable recording and the temporal separation of AE signals during loading. The upper value of the test load was not set, and the test was stopped after the appearance and propagation of visible defects in the tested sample.

#### 2.2.1. AE Sensing System

The AE method is based on the phenomenon of elastic wave generation during the formation and propagation of defects in the tested material. Piezoelectric AE transducers connected to the AE data collection and processing system via a pre-amplification unit are used to record elastic waves. As a result of the action of elastic acoustic waves on piezoelectric transducers (direct piezoelectric effect), an analog electrical signal is generated. The conversion of analog signals into a digital data stream is performed using analog-to-digital converters built into the AE diagnostics system. Due to its high sensitivity, the AE method is one of the most widely used methods of technical diagnostics used in the creation of structural health monitoring systems [43].

The AE sensing system is based on the Vallen AMSY-6 (Vallen Systeme GmbH, Wolfratshausen, Germany) monitoring and processing unit. A quasi-resonant piezoelectric AE transducer VS150-RIC with a built-in 34 dB preamplifier was used as a receiving transducer connected to the AE monitoring unit. The bandwidth of the used AE transducer corresponds to 100–400 kHz, with a maximum sensitivity at a frequency of 150 kHz. The AE transducer (designated as “AE” in Figure 3) was fixed on the steel plate P_2_ with a clamp through a layer of contact grease at a distance of 25 mm from the crystal.

#### 2.2.2. Cross-Polarization Imaging System

The cross-polarization imager consists of an illumination system and a polarization-sensitive camera (Figure 3). The illumination system is the white LED-based wideband light source equipped with a diffusing plate (DP), a collimating Fresnel lens (FL) and a linear polarizer (PF) (laminated film polarizer, extinction ratio 100:1 in a 400–700 nm wavelength range). An image of the specimen illuminated by linearly polarized light is acquired using the polarization-sensitive camera. To reduce the influence of optical rotatory power dispersion, the camera is equipped with combination of colored glass, absorbing bandpass filters (SF) (CWL 540 nm, FWHM 50 nm). The image is formed using the machine vision lens (35 mm focal length, f/1.65) with an additional 3× extender, providing the overall magnification of 64 pixels per 1 mm in the object plane. An image sensor with an on-chip polarizer array (DZK 33UX250, The Imaging Source Europe GmbH, Bremen, Germany) allows for the simultaneous acquisition of four images formed by the light with different polarization plane orientations (0°, 45°, 90° and 135°) in a single frame. During the preliminary adjustment, the camera is aligned with the orientation of the illumination polarization plane provided by the linear polarizer. We analyzed the polarization features of the specimen, considering several criteria calculated using the corresponding single polarization images, *I*_0_*(x,y)*, *I*_45_*(x,y)*, *I*_90_*(x,y)* and *I*_135_*(x,y)*. From the intensity values in the single polarization images, one can calculate the Stokes parameters for the linear polarization case and assess the alterations in the light polarization state introduced by different spatial areas of the inspected sample.

### 2.3. Data Processing

#### 2.3.1. AE Signal Processing

Prior to the compression bench tests, the optimal parameters of the Vallen AMSY-6 unit should be determined. The AE pulse discrimination threshold (*u_th_*) is determined according to the condition *u_th_* ≥ *u_n_* + 6 dB (*u_th_* is the AE pulse discrimination threshold, *u_n_* is the maximum amplitude of noise signals), and, for the described configuration of the experimental setup, it is *u_th_* = 34 dB. To eliminate noise signals arising due to the friction of the UTM upper platen and the crystalline specimen surface, we adjusted the digital filters’ bandwidth for the range Δ*f_p_* = 95–850 kHz. Also, before each bench test, we checked the quality of the acoustic contact between the specimen and the transducer by pressing the graphite pencil lead against the specimen’s side face (Hsu-Nielsen source). The amplitude of the pulses the from pencil lead breakage was in the range of *u_m_* = 99.7–99.8 dB in all of the tests, indicating the low attenuation of the acoustic signals in the AE sensing system.

For the AE monitoring of TeO_2_ single crystals during the compression, we analyzed the primary and complex AE parameters and calculated the high-level quantiles of their empirical distribution functions. The calculation of the empirical distribution functions was carried out using a sliding window function $F_{M}^{*}$ defined as follows:

(1) $$F_{M}^{*}(y)=\frac{1}{M}\sum_{i=1}^{M}I(X_{i}<y),$$

where *M* is a window size, *I* is a quantity of the AE parameters satisfying the condition *X_i_* < *y*, *X_i_* is the AE parameter value from the sample *X = (X_1_, …, X_i_, …, X_M_)* and *y* is a threshold value of the AE parameter in the following range: *y* ∈ [*X_min_, …, X_max_*].

At the first stage of data processing, we assessed the evolution of the primary AE data flow, pulse amplitude *u_m_* and duration *t_imp_*, which we registered during the compression until the visible defect formation occurred. The empirical function of the AE pulse duration distribution may be implemented as the informative diagnostic value indicating the initiation and growth of defects in the TeO_2_ single crystals [44]. Thus, at the second stage of experimental data processing, we propose to consider the high-level quantile of the empirical distribution function of the AE pulse duration *t_imp_*(*p* = 0.85) as a criterion for the detection of the emerging defects using the AE technique.

#### 2.3.2. Cross-Polarization Image Processing

Each 2 × 2-pixel block of a raw image contains four intensity values (*I*_0_, *I*_45_, *I*_90_ and *I*_135_). After single-polarization image extraction, they are used for calculating the following maps: non-polarized (NP), angle of linear polarization (AoLP) and degree of linear polarization (DoLP). The NP map is a monochrome image formed by non-polarized light, calculated as the mean intensity value of the 2 × 2-pixel block:

(2) $$NP=\frac{I_{0}+I_{45}+I_{90}+I_{135}}{4}.$$

Assuming the linear polarization of light after passing through the inspected crystal, the Stokes parameters may be calculated as follows:

(3) $$\begin{array}{l} S_{0}=I_{0}+I_{90},\\ S_{1}=I_{0}-I_{90},\\ S_{2}=I_{45}-I_{135}, \end{array}$$

The *S*_3_ parameter necessary for the circular polarization is not available for this sensor type. However, using the Stokes parameters, values (2) for each pixel block, AoLP and the DoLP spatial maps are derived as follows:

(4) $$\mathrm{AoLP}=\arctan\frac{S_{2}}{S_{1}},$$

(5) $$\mathrm{DoLP}=\frac{\sqrt{S_{1}^{2}+S_{2}^{2}}}{S_{0}}.$$

The AoLP and DoLP spatial maps are helpful for tracking the changes in polarization features. During the experiments, we acquired raw images with resolutions of 1280 × 960 pixels at 40 fps and calculated 640 × 480 pixel NP, AoLP and DoLP maps.

**Figure 4:**
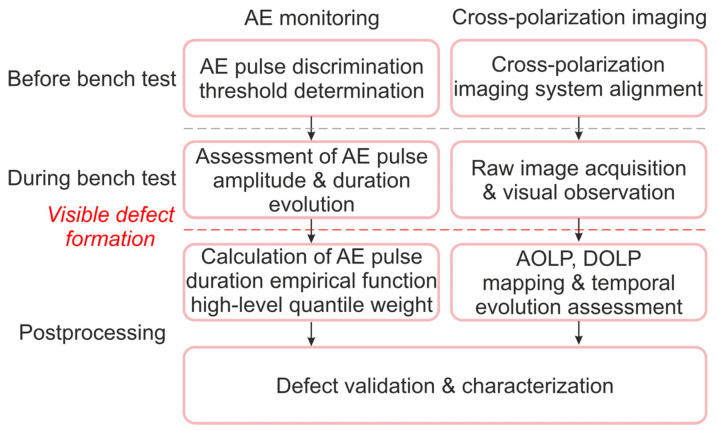
Joint AE and cross-polarization imaging data processing pipeline.

The joint data processing pipeline for simultaneous AE monitoring and cross-polarization imaging is presented in Figure 4.

## 3. Results

### 3.1. AE vs. Optical Data

#### 3.1.1. Compressive Load along the [001] Axis

**Figure 5:**
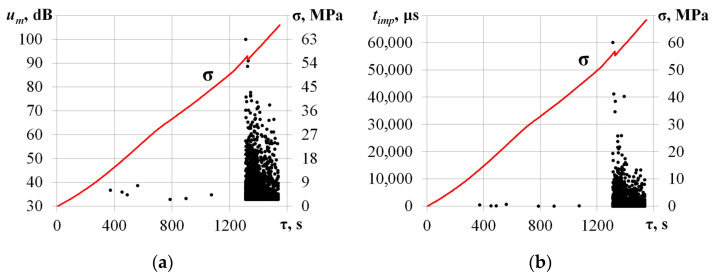
Temporal dependencies of AE pulse amplitude *u_m_* (**a**) and duration *t_imp_* (**b**) time v (dotted) with the compressive stress σ values (solid line) for the sample loaded along the [001] axis.

Figure 5 shows the temporal dependencies of AE pulse amplitude *u_m_* and duration *t_imp_* time and compressive stress σ values for the specimen loaded along the [001] axis.

**Figure 6:**
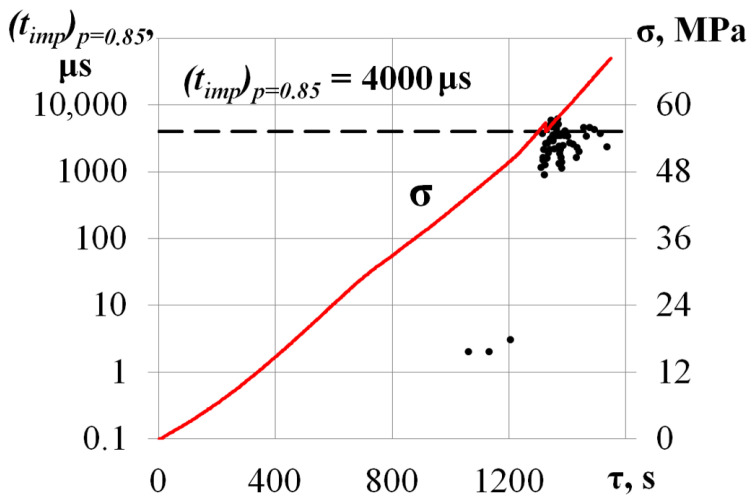
Values belonging the *p* = 0.85 level quantile of the AE pulse duration empirical distribution function (*t_imp_*)*p* = 0.85 for the specimen loaded along the [001] axis.

In the beginning of the compression test (σ ≤ 50 MPa), a low-intensity AE data flow was recorded. The maximum amplitude and duration did not exceed *u_m_* = 40 dB and *t_imp_* = 1000 μs. Further compressive stress increases led to a growth in the values of the primary AE parameters. As the load reached σ = 56.8 MPa, amplitude and duration grew to *u_m_* = 100 dB and *t_imp_* = 60,000 μs. A sharp increase in AE pulse duration may be associated with the friction of the edges of a developing crack during crystal compression, which is confirmed by a decrease in the level of mechanical stress from σ = 56.8 MPa to σ = 55.2 MPa at τ = 1325.4 s. Figure 6 shows the calculated values belonging to the *p* = 0.85 level quantile of the AE pulse duration empirical distribution function.

Before the local decrease in mechanical stress from σ = 56.8 MPa to σ = 55.2 MPa (τ < 1325.4 s), the AE data flow did not exceed (*t_imp_*)*p* = 0.85 = 10 μs. At the same moment (local decrease), a significant growth in the flow ((*t_imp_*)*p* = 0.85 ≥ 4000 μs) was noted.

**Figure 7:**
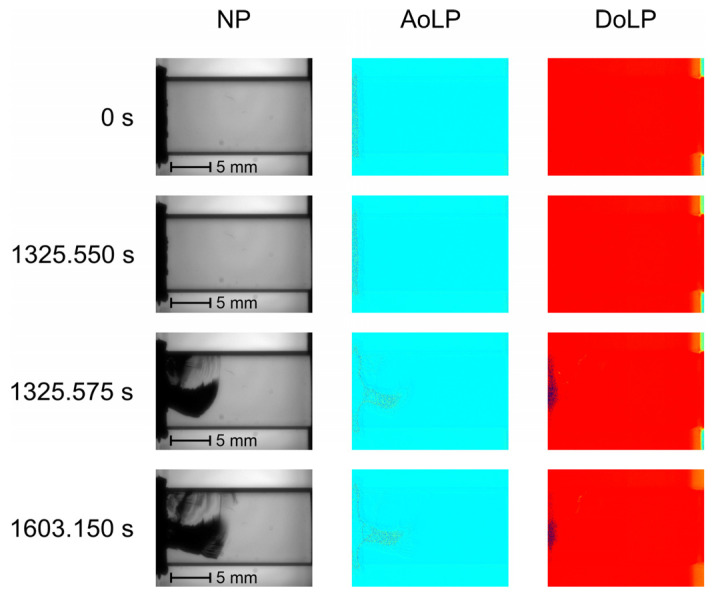
NP, AoLP and DoLP maps of TeO_2_ sample loaded along [001] axis.

Since, for this type of sample, the compressive load is applied along the optical axis [001] with metal platens, optical observation is available only through the polished faces (110) and (1-10). Figure 7 illustrates the typical AoLP and DoLP maps at the most significant defect formation stages. This specimen type conserves the polarization state, and compressive load does not induce changes in the AoLP and DoLP patterns. A defect appears between 1325.55 s and 1325.275 s, as may be seen in NP images. The significant light intensity reduction caused by the crack leads to noise-pattern formation in the damaged zones of the AoLP and DoLP maps. In defect-free areas, AoLP and DoLP values remain stable. The gradual growth of the crack continues until the end of the bench test.

**Figure 8:**
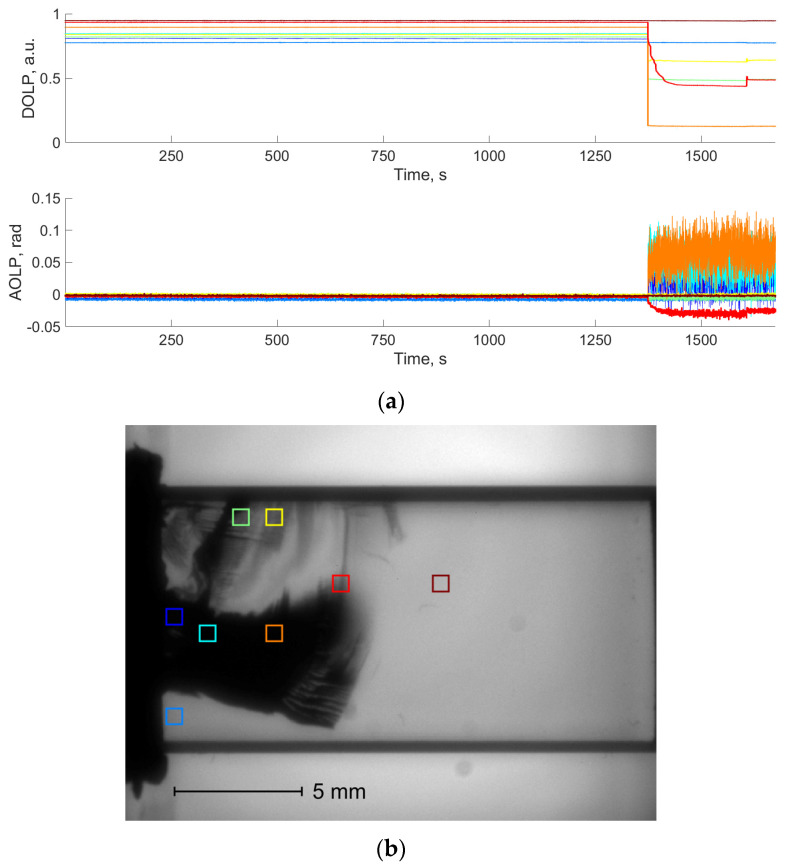
DoLP and AoLP temporal dependencies in TeO_2_ sample loaded along [001] axis (**a**) in selected areas, shown in the NP image acquired at the end of the bench test (**b**). Colors of rectangular areas in (**b**) correspond to curve colors in (**a**).

To analyze the evolution of DoLP and AoLP values during the bench test, the image of the crystal was divided into 20 × 20-pixel areas. In each of them, we calculated average AoLP and DoLP values. Figure 8a demonstrates the AoLP and DoLP temporal dependencies for the areas shown in Figure 8b. AoLP and DoLP across the entire specimen remain stable until the crack appears. After this, individual areas demonstrate significant reductions in DoLP and noise-like AoLP due to the low light intensity in the damaged areas, as mentioned before.

#### 3.1.2. Compressive load along the [1-10] axis

**Figure 9:**
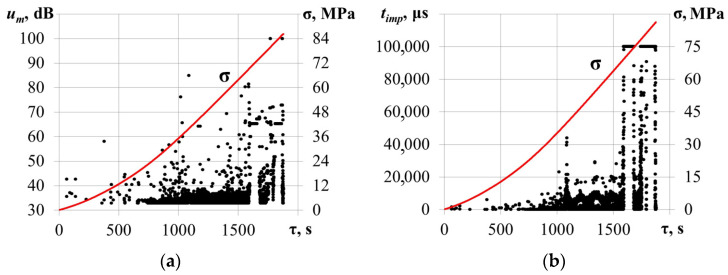
Temporal dependencies (dotted) of AE pulse amplitude *u_m_* (**a**) and duration *t_imp_* (**b**) time, and compressive stress σ values (solid) for the sample loaded along the [1-10] axis.

Figure 9 shows the temporal dependencies of AE pulse amplitude *u_m_* and duration *t_imp_* time, as well as the compressive stress σ values for the TeO_2_ sample loaded along the [1-10] axis.

**Figure 10:**
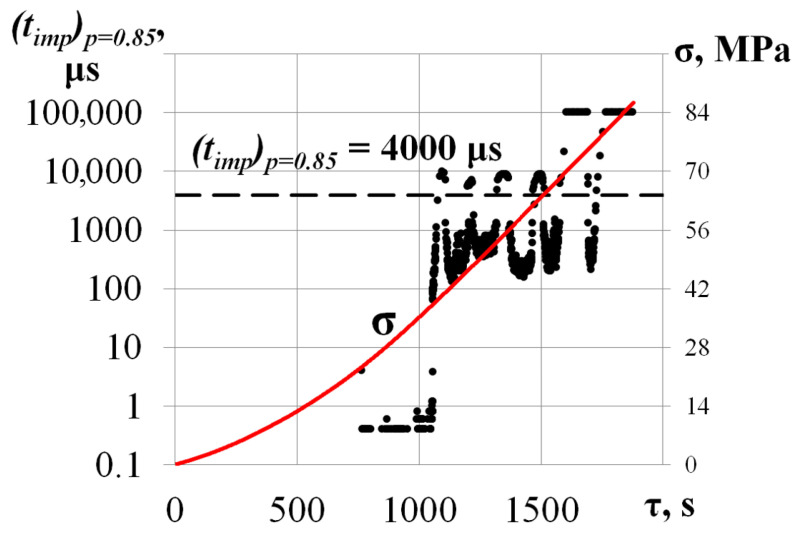
Values belonging the *p* = 0.85 level quantile of the AE pulse duration empirical distribution function (*t_imp_*)*p* = 0.85 for the specimen loaded along the [1-10] axis.

During the compression load, evolution patterns of primary AE parameters appear quite similar to the data acquired for the sample loaded along the [001] axis. The amplitude and duration of AE pulses did not exceed *u_m_* = 45 dB and *t_imp_* = 5000 μs. Due to the friction of the growing crack edges, the amplitude and duration increased sharply at τ = 1087.4 s и τ = 1386.5 s. For the sample loaded along the [1-10] axis, the *p* = 0.85 quantile values of the empirical distribution function of the AE pulse duration are shown in Figure 10.

**Figure 11:**
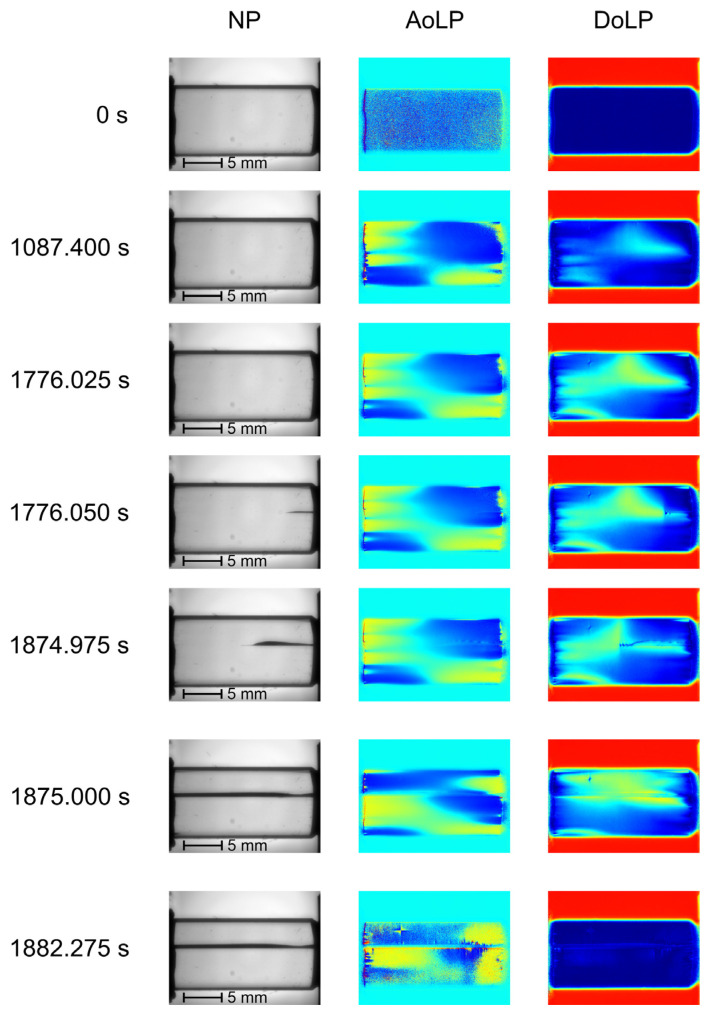
NP, AoLP and DoLP maps of TeO_2_ specimen loaded along [1-10] axis.

Cross-polarization imaging of the samples was carried out along the [001] axis. Thus, the polarization plane orientation ambiguity associated with optical rotatory power dispersion leads to a noise-like pattern in AoLP maps and zero DoLP without external mechanical load, i.e., at t = 0 s. The main stages of crack growth are shown in Figure 11.

As the compression force increases, the DoLP increases in locally stressed zones. In the example shown in Figure 11, the crack-like defect appears in the field of view after 1747 s. Its initial location is highlighted with the edge of increased DoLP near the moving platen. In the short time interval between two consequent frames acquired after 1776.025 s and 1776.05 s, the crack length sharply increases and becomes easily notable in the NP image. Between 1776.05 s and 1874.975 s, the defect grows gradually. After 1875 s, the crack reaches the other platen, and the specimen breaks into two pieces. When the compression load is removed, the residual deformation leads to inhomogeneous AoLP and DoLP maps in both pieces of the sample.

**Figure 12:**
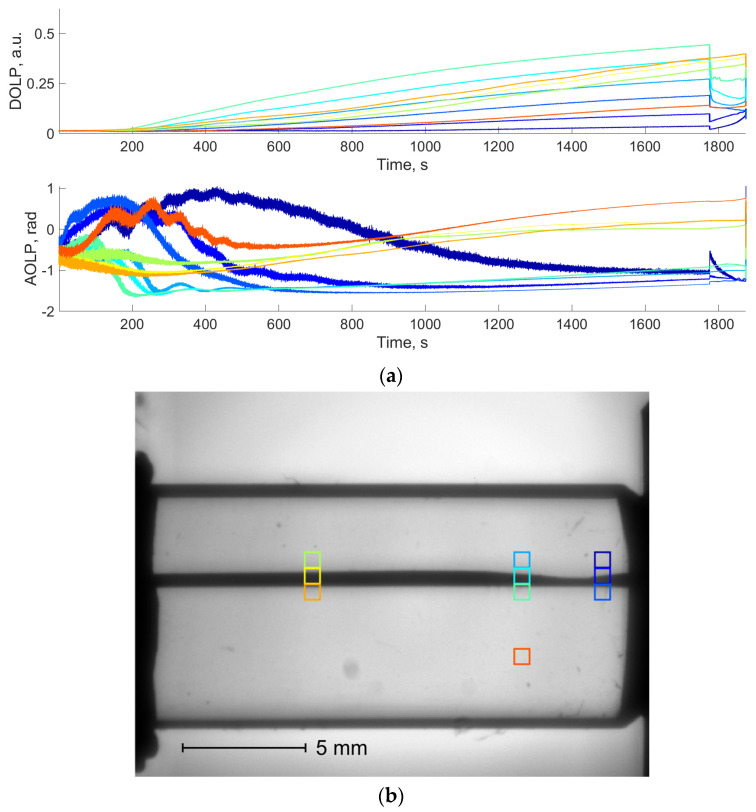
DoLP and AoLP temporal dependencies in TeO_2_ sample loaded along [1-10] axis (**a**) in selected areas shown in the NP image acquired at the end of the bench test (**b**). Colors of rectangular areas in (**b**) correspond to curve colors in (**a**).

Figure 12a demonstrates the temporal dependencies in the selected areas (Figure 12b) located near the initial defect formation zone, at the crack tip after its sharp growth between 1776.025 s and 1776.05 s, far from the area of the initial crack formation and in the undamaged zone. The DoLP in all selected regions progressively increases until 1776.025 s. Then, the response depends on the distance to the area of the initial defect formation.

### 3.2. Quantitative Criteria for Irreversible Damage Initiation and Crack-like Defects Formation

#### 3.2.1. Acoustic Emission

To characterize a crystal damage degree, we propose calculating the weight of the AE data flow meeting the criterion of (*t_imp_*)*p* = 0.85 ≥ 4000 μs (*W*):

(6) $$W=\frac{N_{p}}{N_{\Sigma }}\cdot 100\%,$$

where *N_p_* is a quantity of the AE data flow meeting the criterion (*t_imp_*)*p* = 0.85 ≥ 4000 μs, *N_Σ_* is an overall quantity of the calculated AE data flow.

**Figure 13:**
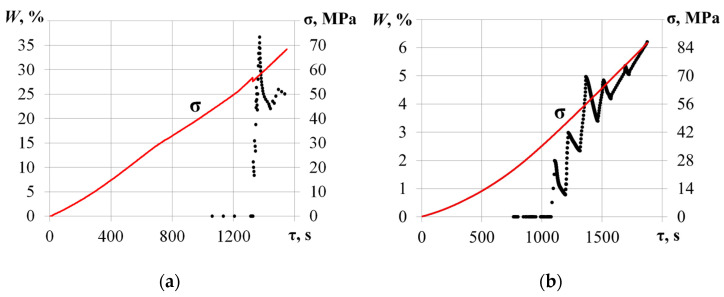
The evolution of AE data flow weight *W* for TeO_2_ samples loaded along [001] (**a**) and [1-10] (**b**) axes.

The examples of the *W* values calculated for two TeO_2_ samples loaded along the [001] and [1-10] axes are presented in Figure 13.

The notable growth in the *W* parameter value at τ = 1325.4 s by 36.7% matches the mechanical stress decrease from σ = 56.8 MPa to σ = 55.2 MPa (Figure 13a). A sharp increase in W calculated with the statistical characteristics of the empirical distribution function of the AE pulse duration is associated with crack edge friction. In Figure 13b, the local minima of *W*(τ) appear at τ = 1087.4 s, and its global maximum appears at the exact moment of the visible complete specimen destruction (τ = 1875 s) and does not exceed 6.2%. The first local maxima of *W*(τ) indirectly point at the initiation of crack-like defect formation before it becomes detectable with the optical imaging techniques. The strong correlation between the intensive crack growth and the *W*(τ) function seems helpful for monitoring the defect development and damage in TeO_2_ crystals.

#### 3.2.2. Cross-Polarization Imaging

**Figure 14:**
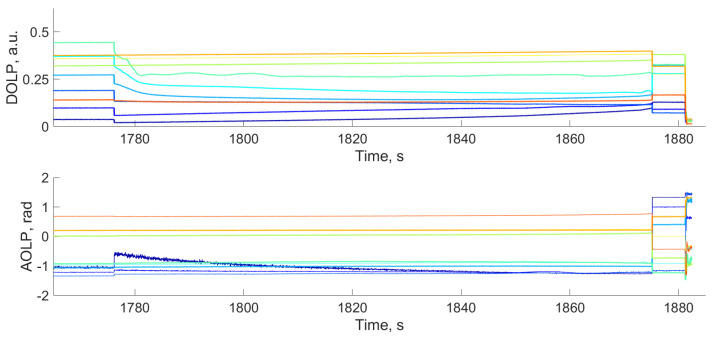
DoLP and AoLP temporal dependencies at the last stage of crack formation in TeO_2_ samples loaded along the [1-10] axis in the areas shown in Figure 12b. Curve colors correspond to to Figure 12.

In comparison to conventional NP images, AoLP and DoLP mapping provides additional data, mainly in the case of observation in the vicinity of the crystal’s optical axis. In contrast with Figure 7, the maps in Figure 11 demonstrate the evolution of the AOLP and DOLP values, indicating the potential zones of defect formation before visible structural changes occur. Also, in this case, the DoLP and AoLP temporal curves at the last stage of crack-like defect formation (Figure 14) show that fast crack growth is accompanied by the sharp changes in these dependencies. These changes may be noticed not only along the crack path, but also in the undamaged areas located in its neighborhood. The impact of the growing defect on the undamaged areas may be associated with the stress distribution in the volume of the sample. In addition, the obtained NP images, as well as the DoLP maps, provide quantitative data on the transmission coefficient and the polarization features posteriori, i.e., after the structural changes happen. Thus, they are not predictive damage indicators but may be informative for AE data validation and the determination of defect location and type.

## 4. Discussion

**Figure 15:**
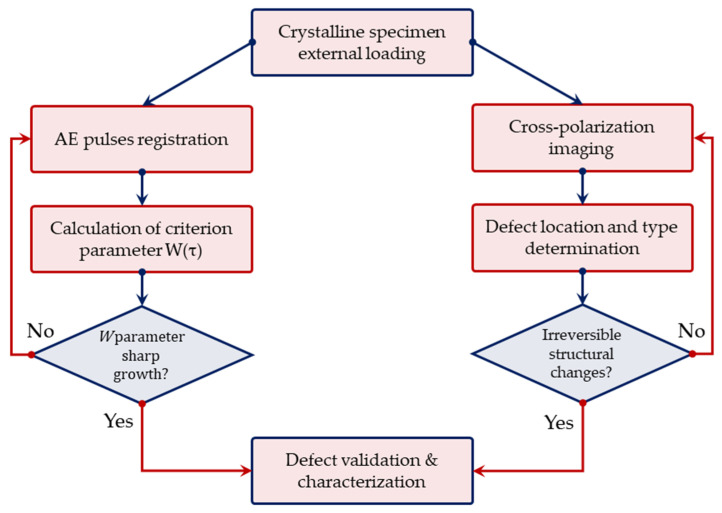
Proposed workflow chart of combined AE sensing and cross-polarization imaging for detection and characterization of crack-like defects in TeO_2_ crystals.

We addressed the issues of determining the informative AE signals specific for defect formation and growth in anisotropic crystals and assessed the applicability of cross-polarization imaging to localize the sample’s areas of increased mechanical stress in situ. We studied the feasibility of joint AE and cross-polarization imaging for monitoring defect formation in anisotropic crystals with different orientations of crystallographic axes without destruction. In contrast to previous AE studies of anisotropic crystals, we carried out continuous crack growth experiments with TeO_2_ crystals of two different crystallographic orientations. We supposed that the weight content of the high-level quantile of the empirical duration distribution function (*W*(τ): (*t_i_*)*p* = 0.85 ≥ 4000 μs) may be an informative AE parameter, as its dynamic change correlates well with the degree of crystal damage. The moments of crack-like defect appearance in the tested samples determined by AE sensing were precisely confirmed by the results of cross-polarization imaging. The applicability of cross-polarization imaging for the localization of increased mechanical stress depends on the orientation of sample crystallographic axes. However, this mapping technique may be implemented for defect location and characterization, regardless of the sample type. In practice, the combination of these two techniques may become an effective approach to the non-destructive testing of crystals in the real operational conditions of crystal-based devices. This is especially important for devices operating under increased quasi-static, cyclic, vibrational or impact mechanical stresses. A possible workflow chart illustrating this concept is shown in Figure 15. Generally, the AE sensing technique provides in situ information on the presence of damage in the studied crystal, and cross-polarization imaging allows us to verify and quantify it, i.e., determine the defect type, location and dimensions.

The dynamics of the changes in the AE parameters and the function *W*(τ) during AE sensing led us to the assumption that not only are the moments of initial crack formation recorded, but so are the friction processes of the crack faces. Therefore, the proposed technique can be used both to study newly manufactured crystals and to control samples that have already been in use for some time to identify existing (residual) damage that appeared during operation.

## 5. Conclusions

In this feasibility study, we found that AE data acquisition and analysis may provide an effective quantitative merit for crack appearance and development under dynamic loading in TeO_2_ crystals widely used in acousto-optics. From the experiments with crystal samples of two different configurations, we established an informative AE parameter—the weight of the high-level quantile in the duration distribution function. The moment of formation and the development of a crack-like defect, according to the results of AE diagnostics, is accompanied by the registration of a local decrease in the level of mechanical stress and an increase in the duration of AE pulses. A sharp increase in the duration of the recorded signals may be due to the process of friction on the edges of a developing crack during compression. Additionally, we verified the results by optical techniques: conoscopy and cross-polarization imaging. The obtained results reveal the patterns specific for these processes and open up new possibilities for the non-destructive real-time monitoring of the structure of optically transparent crystals under their operating conditions. The prospective studies include monitoring anisotropic crystals of various types using the proposed workflow under lower compressive loads without defect appearance and development.

## Data Availability

Experimental data are available from the corresponding author upon reasonable request.
